# ALPS-Like Phenotype Caused by ADA2 Deficiency Rescued by Allogeneic Hematopoietic Stem Cell Transplantation

**DOI:** 10.3389/fimmu.2018.02767

**Published:** 2019-01-14

**Authors:** Federica Barzaghi, Federica Minniti, Margherita Mauro, Massimiliano De Bortoli, Rita Balter, Elisa Bonetti, Ada Zaccaron, Virginia Vitale, Maryam Omrani, Matteo Zoccolillo, Immacolata Brigida, Maria Pia Cicalese, Massimo Degano, Michael S. Hershfield, Alessandro Aiuti, Anastasiia V. Bondarenko, Matteo Chinello, Simone Cesaro

**Affiliations:** ^1^Pediatric Immunohematology and Bone Marrow Transplantation Unit, IRCCS San Raffaele Scientific Institute, Milan, Italy; ^2^San Raffaele Telethon Institute for Gene Therapy, IRCCS San Raffaele Scientific Institute, Milan, Italy; ^3^Department of Systems Medicine, Tor Vergata University, >Rome, Italy; ^4^Paediatric Hematology-Oncology, Ospedale della Donna e del Bambino, Verona, Italy; ^5^Biocrystallography Unit, Division of Immunology, Transplantation and Infectious Diseases, IRCCS San Raffaele Scientific Institute, Milan, Italy; ^6^Department of Medicine and Biochemistry, Duke University School of Medicine, Durham, NC, United States; ^7^Vita-Salute San Raffaele University, Milan, Italy; ^8^Department of Pediatric Infectious Diseases and Immunology, Medical Academy of Postgraduate Education, Kiev, Ukraine

**Keywords:** ADA2 deficiency, HSCT, ALPS, ADA2, CECR1, autoimmunity, immunodeficiency, neutropenia

## Abstract

Adenosine deaminase 2 (ADA2) deficiency is an auto-inflammatory disease due to mutations in cat eye syndrome chromosome region candidate 1 (*CECR1*) gene, currently named *ADA2*. The disease has a wide clinical spectrum encompassing early-onset vasculopathy (targeting skin, gut and central nervous system), recurrent fever, immunodeficiency and bone marrow dysfunction. Different therapeutic options have been proposed in literature, but only steroids and anti-cytokine monoclonal antibodies (such as tumor necrosis factor inhibitor) proved to be effective. If a suitable donor is available, hematopoietic stem cell transplantation (HSCT) could be curative. Here we describe a case of ADA2 deficiency in a 4-year-old Caucasian girl. The patient was initially classified as autoimmune neutropenia and then she evolved toward an autoimmune lymphoproliferative syndrome (ALPS)-like phenotype. The diagnosis of ALPS became uncertain due to atypical clinical features and normal FAS-induced apoptosis test. She was treated with G-CSF first and subsequently with immunosuppressive drugs without improvement. Only HSCT from a 9/10 HLA-matched unrelated donor, following myeloablative conditioning, completely solved the clinical signs related to ADA2 deficiency. Early diagnosis in cases presenting with hematological manifestations, rather than classical vasculopathy, allows the patients to promptly undergo HSCT and avoid more severe evolution. Finally, in similar cases highly suspicious for genetic disease, it is desirable to obtain molecular diagnosis before performing HSCT, since it can influence the transplant procedure. However, if HSCT has to be performed without delay for clinical indication, related donors should be excluded to avoid the risk of relapse or partial benefit due to a hereditary genetic defect.

## Introduction

Deficiency of adenosine deaminase 2 (DADA2) is a genetic auto-inflammatory disease caused by autosomal recessive mutations in adenosine deaminase 2 (*ADA2*) gene, mapped on chromosome 22q11 (1). Adenosine deaminase 2 (ADA2) is an enzyme secreted in the plasma by myeloid cells that catalyzes the conversion of adenosine to inosine (2). The pathomechanism of DADA2 remains unclear, but immunomodulatory function of ADA2 is crucial. Indeed, *in vitro* studies suggest that ADA2 works as an autocrine factor activating cells of the immune system (3). Thus, deficiency of ADA2 results in multiple immune abnormalities, such as increased pro-inflammatory M1 macrophages (as opposed to anti-inflammatory M2 macrophages), upregulation of genes associated with neutrophil activation, increased secretion of pro-inflammatory cytokines including TNF and interleukin-1b (IL-1b) and endothelial damage (4, 5).

DADA2 was described for the first time in 2014 by two independent studies (4, 6). The predominant clinical presentation includes recurrent fever, early-onset vasculopathy involving skin, gut and central nervous system (1, 7, 8). Blood tests usually show elevation of acute phase reactants (erythrocyte sedimentation rate and C-reactive protein) (1). Recent evidences suggest a broader clinical spectrum encompassing also features of autoimmunity, immunodeficiency and bone marrow dysfunction (1, 8–14). The diagnosis of DADA2 is based on the measurement of plasma ADA2 enzymatic activity and on the identification of biallelic mutations in *ADA2*.

Splenomegaly or hepatosplenomegaly are frequently reported in patients with DADA2 deficiency (1, 4, 11, 15, 16) as well as cytopenia (1, 4, 11, 17). Nevertheless, their presence in addition to clinical and biological features defining an Autoimmune Lymphoproliferative Syndrome (ALPS)-like phenotype is less common. Only two cases of DADA2 presenting with clinical features resembling ALPS have been recently described (17, 18). Another patient experienced hemophagocytic lymphohistocytosis on a background of ALPS-like manifestations (19).

We describe a case of ADA2 deficiency presenting as ALPS-like disease, unresponsive to immunosuppressive therapy, successfully treated with allogeneic hematopoietic stem cell transplantation (HSCT).

## Case Presentation

A 4-year old Caucasian female child was referred to our Center for diagnostic assessment of severe neutropenia lasting for 6 months that was not responsive to the administration of granulocyte-colony stimulating factor (G-CSF) up to a maximum dose of 20 mcg/kg daily.

The patient was born after a full-term gestation, from non-consanguineous parents. The birth weight was 3,600 g. Familial medical history was unremarkable. Parents reported recurrent infections of upper respiratory tract and stomatitis during the first years of life, before the occurrence of severe neutropenia.

Physical examination was normal, except for the presence of splenomegaly (long axis 11.5 cm at ultrasound scan), whereas blood count revealed profound neutropenia (absolute neutrophil count, ANC, 0.02 × 10^9^/L) without anemia and thrombocytopenia. Bone marrow examination showed the presence of rare myeloid precursors with normal representation of the erythroid and megakaryocyte lineages. The clonogenic study of bone marrow progenitors *in vitro* showed a normal growth of erythroid cells whereas the growth of myeloid cells was below the normal range: CFU-E/BFU-E 37 (normal range 27–81/2 × 10^4^); CFU-GEMM 1 (normal range 0–10/2 × 10^4^); CFU-GM 30 (normal range 33–100/2 × 10^4^). Interestingly the patient bone marrow plasma markedly inhibited the growth of progenitor cells obtained from healthy voluntary bone marrow donors: CFU-E/BFU-E/(2 × 10^5^) from 88 (controls) to 4 (controls + patient plasma), CFU-GEMM/(2 × 10^5^) from 17 to 9. An extensive diagnostic work-up excluded other potential causes of neutropenia such as Kostmann disease, Shwachman-Diamond syndrome, Fanconi anemia and paroxysmal nocturnal hemoglobinuria. Finally, a misdiagnosed form of autoimmune neutropenia was hypothesized. Indeed, anti-neutrophil IgG were 171.1 mg/dL (normal value < 33.5 mg/dL), IgM were 24.9 mg/dL (normal value < 32.2 mg/dL). Hence, the patient underwent a 4-week trial with prednisone at 2.5 mg/kg/day that led to complete remission of neutropenia (ANC 3.360 × 10^9^/L). Prednisone was slowly tapered. Nevertheless, fever occurred at every attempt of reducing the dose of prednisone below 1 mg/kg/day. Given the significant side effects after 3 months of steroid therapy, introduction of steroid-sparing agents (cyclosporine first and mycophenolate mofetil later) was attempted. However, neutropenia did not improve and the patient remained steroid dependent.

After 4 months, she experienced a single episode of livedo reticularis at the lower limbs. The histological examination showed a livedoid thrombotic vasculopathy. Moreover, the patient was hospitalized eight times due to episodes of fever, abdominal pain and neutropenia with elevated inflammation markers. Abdominal CT scan, esophagogastroduodenoscopy (EGDS) and colonoscopy showed no abnormalities, as well as gastrointestinal biopsies.

The patient was assessed also for ALPS (Table 1) (20) because of autoimmune neutropenia and splenomegaly: she displayed increased double negative T-lymphocytes in the peripheral blood (2.5%, normal value <1.7% of total lymphocytes), IL-18 (1.125 pg/ml, normal value 36–258 pg/ml), IL-10 (7.2 pg/ml, normal value < 1 pg/ml). However, vitamin B12 levels where only mildly elevated as compared to patients with classical ALPS (775 pg/mL, normal value 191–663) (21); similarly, FAS-induced apoptosis test was normal (survivor T-lymphocytes after FAS antigenic stimulation 42%) (22). Sirolimus was started (23, 24) and withdrawn after 2 months, due to the occurrence of fever and abdominal pain. Prednisone was continued alone. No mutations were found in ALPS-disease causative genes (*FAS, FASL, CAS10*). The control of clonogenic tests 7 months after starting immunosuppression showed a good growth of CFU-E/BFU-E (120/*2* × *10*^4^*)* and CFU-GEMM (*52/*2 × 10^4^); the growth of bone marrow progenitor cells derived from healthy donors was no more inhibited by the patient's bone marrow plasma.

**Table 1:** Diagnostic criteria for ALPS defined by 2009 NIH consensus (20).

**Required**
1. Chronic (6 months), nonmalignant, noninfectious lymphadenopathy or splenomegaly or both
2. Elevated CD3 + TCRαβ + CD4-CD8- DNT cells (1.5% of total lymphocytes or 2.5% of CD3 lymphocytes) in the setting of normal or elevated lymphocyte counts
**Accessory**
***Primary***
1. Defective lymphocyte apoptosis (in 2 separate assays)
2. Somatic or germline pathogenic mutation in FAS, FASL, or CASP10
***Secondary***
1. Elevated plasma sFASL levels (>200 pg/mL) OR elevated plasma IL-10 levels (>20 pg/mL) OR elevated serum or plasma vitamin B12 levels (>1,500 ng/L) OR elevated plasma IL-18 levels (>500 pg/mL)
2. Typical immunohistological findings as reviewed by an experienced hematopathologist
3. Autoimmune cytopenias (hemolytic anemia, thrombocytopenia, or neutropenia) AND elevated immunoglobulin G levels (polyclonal hypergammaglobulinemia)
4. Family history of a nonmalignant/noninfectious lymphoproliferation with or without autoimmunity

*Definitive diagnosis: required criteria plus one primary accessory criterion*.

*Probable diagnosis: required criteria plus one secondary criterion*.

*CASP10 caspase 10, DNT double-negative T cell, FASL Fas-ligand, Ig immunoglobulin, IL interleukin, NIH National Institutes of Health, sFasL soluble Fas ligand*.

Considering the persistence of neutropenia despite the administration of G-CSF and immunosuppressive therapy, the patient was candidate to allogeneic HSCT. During pre-transplant workup, brain magnetic resonance (MRI) revealed a 5-mm signal alteration in the right cortical-subcortical frontal basal region consistent with a petechial spot.

HSCT was performed at 5 years and 4 months of age. The patient underwent a myeloablative conditioning with busulfan, thiotepa, fludarabine and rituximab (Table 2). Graft-vs.-host disease (GVHD) prophylaxis consisted of anti-thymocite globulin, short methotrexate, cyclosporine (from day 0 to day +28) than replaced by tacrolimus (from day +29). Neutrophil engraftment occurred on day +26, and platelet engraftment occurred on day +34. Complete donor chimerism was detected on day +33. Main complications after HSCT were gut and skin grade II acute GVHD requiring additional therapy with prednisone at 2 mg/kg/day, tapered slowly due to frequent relapses. Further complications were: CMV reactivation (on day + 31) treated successfully with valganciclovir and osteonecrosis of the knees (on day + 138), treated with vitamin D, calcium, bisphosphonates, physio-kinesiotherapy and hyperbaric oxygen therapy.

**Table 2:** Conditioning regimen.

**Drug**	**Dosage**
Fludarabine	3 × 50 mg/m^2^/day
Thiotepa	2 × 5 mg/kg/day
Busulfan	16 × 0.8 mg/kg (every 6 h)
ATG Genzyme	3 × 4 mg/kg/day
Rituximab	1 × 375 mg/m^2^/day

Considering the atypical clinical evolution, the inadequate response to therapy and the absence of mutations, the diagnosis of ALPS was questioned. We performed molecular analysis by Haloplex target system platform (Illumina) on the patient's buccal swab obtained after HSCT. The patient resulted compound heterozygous for two novel mutations in *ADA2* gene. The variant located at exon 9 determines an amino acidic substitution (c.1367 A>G, p.Y456C) and the variant located in exon 8 introduces a premature stop codon causing the synthesis of a truncated protein (c.1196. G>A, p.W399^*^). Both parents were healthy heterozygous carriers. These new mutations are reported as detrimental for the protein according to different prediction tools (e.g., PROVEAN, PolyPhen).

**Figure 1:**
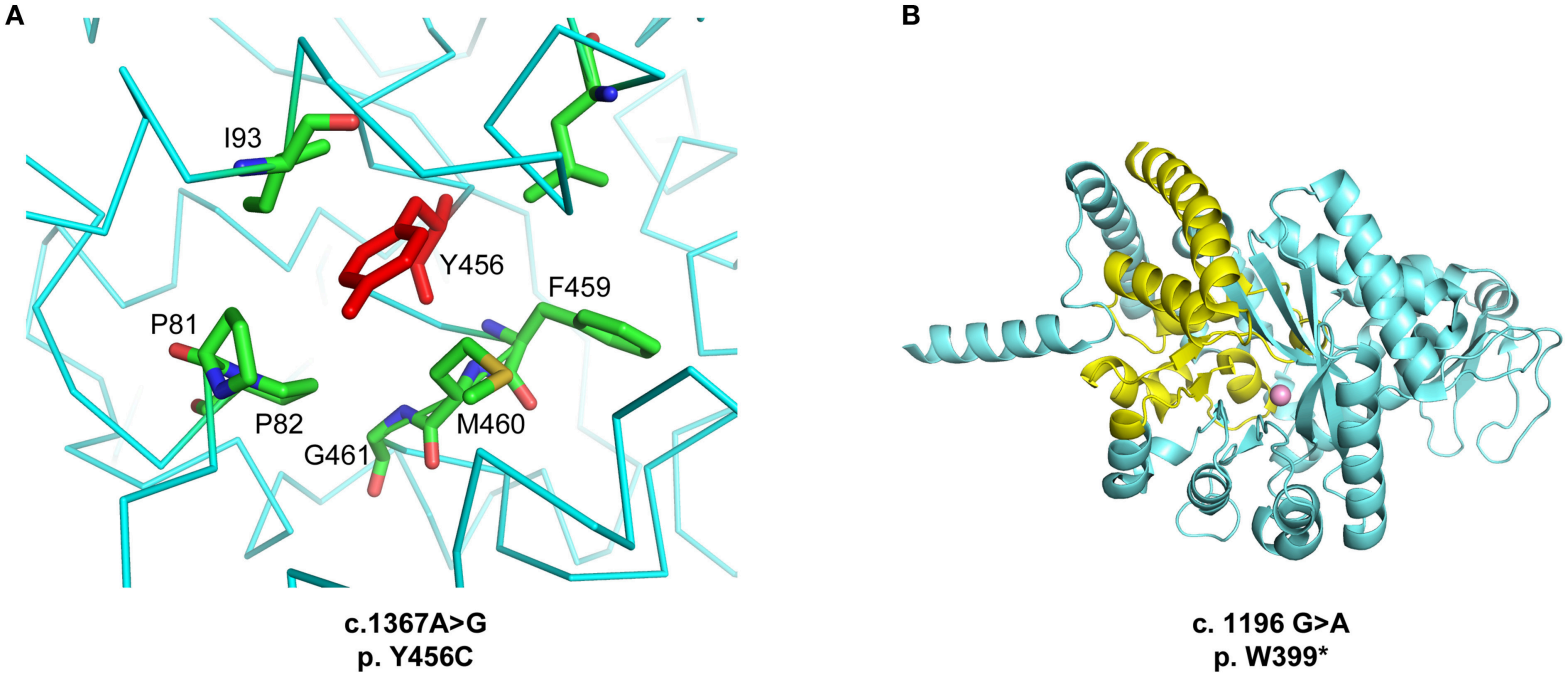
**(A)** Location of the p.Y456C mutation. The Y456 residue is located in an α-helical segment and inserted between hydrophobic residues. **(B)** Structural abnormality of the ADA2 protein generated by the p.W399* mutation. In yellow, the portion of the protein that is lacking due to the stop codon introduced by the mutation. Figures generated with Pymol (http://www.pymol.org).

The high-resolution crystal structure of the homodimeric wild type protein was previously determined (PDB ID 3LGD, corresponding to UniProt Q9NZK5 amino acid residues 29-510, Zavialov et al.,http://dx.doi.org/10.1074/jbc.M109.083527). To address the effects of the two mutations, we performed a structural and energetic analysis of the enzyme. The p.Y456C mutation affects a hydrophobic pocket of ADA2 (Figure 1A). Of note, this is a novel mutation but the p.Y453C mutation, which affects the same hydrophobic pocket, is one of the most common found in patients with DADA2 (1). The p.Y456C mutation carried by our patient heavily destabilizes the ADA2 structure (ΔΔ G = 4.53 ± 0.12 kcal/mol from n = 5 independent simulations) as calculated using the program FoldX (25). The p.W399^*^ mutation instead leads to a truncation of the protein C-terminus, removing key residues required for the correct folding of the active site, and resulting in a solvent-exposed catalytic Zn^2+^ ion (Figure 1B). Thus, both mutations are predicted to dramatically reduce the enzymatic activity of ADA2 through structural destabilization of the protein.

Pre-HSCT plasma of the patient was not available but we could assess ADA2 activity in the parents, which resulted lower than healthy controls as expected in carriers, supporting the deleterious effect of the mutations on the protein function. Moreover, patient's ADA2 plasma activity after HSCT was normal, in the absence of clinical manifestations of the disease and in the presence of full donor chimerism (Table 3). These results strongly suggest DADA2 as cause of the hematological manifestations of the patient.

**Table 3:** Plasma ADA2 activity in the index family.

**Sample**	**Age (year)**	Plasma ADA2 activity^*^ (mU/mL)
Patient post-HSCT	7	21.4
Mother	32	4.2
Father	36	4.9
Reference values for plasma ADA2 activity^*^ **(mU/mL), mean** ± **SD (range)**		
ADA2 deficient (*n* = 36)		0.4 ± 0.5 (0.02 – 1.9)
ADA2 carriers (*n* = 25)		5.5 ± 1.7 (2.9 – 8.8)
Controls (*N* = 23 + pooled human plasma)		13.8 ± 5.1 (4.8 – 27.2)

* *obtained using the HPLC assay described in Zhou et al. (4)*.

The patient gradually recovered from HSCT complications and immunosuppressive therapy was withdrawn. She is now alive and well 28 months after HSCT enjoying a normal life.

## Discussion

Clinical phenotype and age at onset of DADA2 vary widely without clear correlation to *ADA2* genotype (8).

Although not described initially, hematological disorders appear to be an emergent clinical manifestation of DADA2. A various degree of hematological dysfunction affects approximately 43% of patients, in the absence of evident vasculitis (14). Anemia, including pure red cell aplasia, and lymphopenia are the most frequent findings, while neutropenia is a rare clinical manifestation (1, 13, 14).

Initially, our patient was classified as an ALPS-like based on autoimmune neutropenia, splenomegaly and positive biomarkers. Subsequently, the diagnosis of ALPS became less certain because of the lack of response to immunosuppressive drugs, atypical clinical features (livedo reticularis and recurrent severe abdominal pain), normal FAS-induced apoptosis test, and lack of mutations of ALPS-related genes. Only widening the genetic evaluation after HSCT in the index case and in the parents, DADA2 was diagnosed. Of note, clinical presentation of DADA2 as ALPS-like disease have been reported in two patients (17, 18) who were successfully treated with HSCT and anti-TNF medications respectively.

Of note, during the active phase of the disease, the patient's bone marrow plasma markedly inhibited the growth of progenitor cells obtained from healthy bone marrow donors. This finding and the reversion of inhibition after administration of steroids, suggests the presence of pro-inflammatory mediators inhibiting the growth of bone marrow progenitor cells, as observed in the setting of acquired aplastic anemia, where plasma pro-inflammatory cytokines block the differentiation of bone marrow progenitor cells (26). This further supports the possible benefit derived by the administration of TNF-inhibitors in ADA2 patients before transplant, in order to reduce the inflammatory burden also in the bone marrow niches (14). Current literature suggests different therapeutic options for DADA2: the choice between medical therapy and HSCT depends on predominant symptoms and severity of the clinical phenotype. Being an inflammatory condition, high doses of steroids are usually useful to control the disease, although a steroid-dependence often occurs (5), as in the case herein described. If inflammatory symptoms and vasculitis are predominant, anti–tumor necrosis factor agents are usually beneficial (6, 7, 11, 14). HSCT is a potential definitive treatment, since it provides ADA2 producing monocytes, which are able to restore ADA2 plasma activity and control the disease manifestations (15, 17, 19, 27). Moreover, as recently reported by Hashem et al. (28), bone marrow dysfunction and immunodeficiency are the most frequent indications leading to transplant.

Our experience suggests that ADA2 deficiency should be considered as differential diagnosis in cases of severe autoimmune neutropenia evolving to ALPS-like disease not responsive to standard immunosuppressive approaches. Early diagnosis based on these manifestations could allow prompt therapeutic intervention anticipating the onset of vasculopathy and related severe complications.

Importantly, clinicians should aim at obtaining the molecular diagnosis of putative genetic diseases before transplant, since it could influence the management of patients candidate to HSCT. This particularly applies in ADA2 deficiency, considering the high risk of complications due to the persisting inflammation and the impaired endothelial integrity (17, 19, 29). In this light, anti-TNF therapy administered before the conditioning can reduce vascular inflammation (14). With the same aim, conditioning should be tailored, for example replacing Busulfan with Treosulfan or other agents displaying lower endothelial toxicity. Another strategy can foresee prophylactic Defibrotide to reduce the risk of sinusoidal obstruction syndrome. In addition, the selection of the donor can favor unrelated donor avoiding carrier familial donors who might display partial reduction of ADA2 activity. This partial defect can be amplified in the presence of mixed chimerism, leaving a residual and potentially clinically significant enzymatic defect (28). Finally, evaluating ADA2 activity after transplant, especially in case of mixed chimerism, allows monitoring the efficacy and promptly detect the disease recurrence (27). Further studies are needed to define the appropriate timing of HSCT and as well as intensity of conditioning regimen.

## Concluding Remarks

Early diagnosis of DADA2 in cases presenting with hematological manifestations, rather than classical vasculopathy, is crucial, allowing the patients to undergo HSCT, if indicated, before the arousal of more severe manifestations of the disease. Moreover, physicians can adjust the transplant procedure based on the features of the disease, in order to minimize complications and create the most favorable conditions to allow an efficacious control of the disease. Finally, this case highlights the importance of reaching a genetic diagnosis before HSCT. If transplant cannot be delayed, a related donor should be excluded in all cases of genetic disease suspicion without precise molecular diagnosis: an unrelated donor would give the possibility to avoid relapse or partial benefit due to a common genetic defect.

## Ethics Statement

The study was performed in accordance with the Declaration of Helsinki. The patient was enrolled in a study approved by the San Raffaele Ethical Committee (TIGET06). Written informed consent was obtained from parents for biological sample collection and data publication.

## Author Contributions

FM, MM, MC, MDB, RB, EB, AZ, VV, and SC participated to clinical care of the patient and related clinical data. IB, MZ, and MO performed the molecular analysis by Haloplex. IB, MZ, MO, FB, and MPC contributed to the selection of variants. MD analyzed the effect of mutations on the protein structure. MH performed the dosage of ADA2 enzymatic activity. FB, FM, MM, AA, and SC designed the study, analyzed results and wrote the paper. AVB referred the patient and participated in the clinical management.

### Conflict of Interest Statement

The authors declare that the research was conducted in the absence of any commercial or financial relationships that could be construed as a potential conflict of interest.
